# The newly synthesized anticancer drug HUHS1015 is useful for treatment of human gastric cancer

**DOI:** 10.1007/s00280-014-2661-z

**Published:** 2015-01-08

**Authors:** Yoshiko Kaku, Ayako Tsuchiya, Takeshi Kanno, Shuhei Nakao, Tadashi Shimizu, Akito Tanaka, Tomoyuki Nishizaki

**Affiliations:** 1 Division of Bioinformation, Department of Physiology, Hyogo College of Medicine, 1-1 Mukogawa-cho, Nishinomiya, 663-8501 Japan; 2 Laboratory of Chemical Biology, Advanced Medicinal Research Center, Hyogo University of Health Sciences, 1-3-6 Minatojima, Chuo-ku, Kobe, 650-8530 Japan

**Keywords:** HUHS1015, Anticancer drug, Gastric cancer, Apoptosis, Suppression of tumor growth

## Abstract

Naftopidil is clinically for treatment of benign prostate hyperplasia, and emerging evidence has pointed to its anticancer effect. To obtain the anticancer drug with the potential greater than that of naftopidil, we have newly synthesized the naftopidil analogue HUHS1015. The present study investigated the mechanism underlying HUHS1015-induced apoptosis of human gastric cancer cells and assessed the possibility for clinical use as an innovative anticancer drug. HUHS1015 reduced cell viability for MKN28 human well-differentiated gastric adenocarcinoma cell line and MKN45 human poorly differentiated gastric adenocarcinoma cell line in a concentration (0.3–100 μM)-dependent manner more effectively than cisplatin, a chemo-drug widely used. In the flow cytometry using propidium iodide (PI) and annexin V, HUHS1015 significantly increased the population of PI-positive and annexin V-negative cells, corresponding to primary necrosis and that of PI-positive and annexin V-positive cells, corresponding to late apoptosis/secondary necrosis, both in the two cell types. HUHS1015 significantly activated caspase-3, caspase-4, and caspase-8 in MKN45 cells, while no obvious caspase activation was found in MKN28 cells. HUHS1015 upregulated expression of the tumor necrosis factor α (TNFα) mRNA and protein in MKN45 cells, allowing activation of caspase-8 through TNF receptor and the effector caspase-3. HUHS1015 clearly inhibited tumor growth in mice inoculated with MKN45 cells, with the survival rate higher than that for the anticancer drugs cisplatin, paclitaxel, and irinotecan. The results of the present study show that HUHS1015 induces caspase-independent and caspase-dependent apoptosis of MKN28 and MKN45 human gastric cancer cells, respectively, and effectively suppresses MKN45 cell proliferation.

## Introduction

Naftopidil, an α_1_-adrenoceptor blocker, is clinically used for treatment of benign prostate hyperplasia and hypertension. Interestingly, naftopidil exhibits an anticancer effect on human bladder, prostate, and renal cancer cells [1]. In our earlier study, naftopidil induced apoptosis of human malignant pleural mesothelioma cells, regardless of α_1_-adrenoceptor blocking [2]. To obtain the more potential anticancer drug, we have newly synthesized 21 naftopidil analogues and assessed the anticancer effect of these compounds. Among them the most expected hit was 1-[2-(2-Methoxyphenylamino)ethylamino]-3-(naphthalene-1-yloxy)propan-2-ol (HUHS1015) [3]. HUHS1015 induces apoptosis of a wide variety of cancer cell types; human malignant mesothelioma cell lines MSTO-211H, NCI-H28, NCI-H2052, and NCI-H2452 cells; human lung cancer cell lines A549, SBC-3, and Lu-65 cells; human hepatoma cell lines HepG2 and HuH-7 cells; human gastric cancer cell lines MKN28 and MKN45 cells; human bladder cancer cell lines 253 J, 5637, KK-47, TCCSUP, T24, and UM-UC-3 cells; human prostate cancer cell lines DU145, LNCaP, and PC-3 cells; and human renal cancer cell lines ACHN, RCC4-VHL, and 786-O cells [3]. Moreover, HUHS1015 induces apoptosis of malignant pleural mesothelioma cells by activating caspase-4 and the effector caspase-3, in part as mediated through a mitochondrial pathway [4].

The present study was conducted to understand the mechanism underlying HUHS1015-induced apoptosis of MKN28 and MKN45 human gastric cancer cells and assess the possibility for clinical use as an effective anticancer drug. We show here that HUHS1015 induces caspase-independent and caspase-dependent apoptosis of MKN28 and MKN45 cells, respectively, and suppresses tumor growth in mice inoculated with MKN45 cells, with the survival rate higher than that for anticancer drugs clinically used.

## Materials and methods

### Animal care

All procedures have been approved by the Animal Care and Use Committee at Hyogo College of Medicine and were in compliance with the National Institutes of Health Guide for the Care and Use of Laboratory Animals.

### Cell culture

Human gastric cancer cell lines MKN28 and MKN45 cells, gifted from Dr. Tatematsu (Nagoya University, Japan), were grown in RPMI 1640 medium supplemented with 10 % (v/v) heat-inactivated fetal bovine serum, penicillin (final concentration, 100 U/ml), and streptomycin (final concentration, 0.1 mg/ml), and cells were incubated in a humidified atmosphere of 5 % CO_2_ and 95 % air at 37 °C.

### Cell viability

Cell viability was evaluated by the method using 3-(4,5-dimethyl-2-thiazolyl)-2,5-diphenyl-2H-tetrazolium bromide (MTT).

### Apoptosis assay

Before and after treatment with HUHS1015, cells were suspended in a binding buffer and stained with both propidium iodide (PI) and annexin V-FITC, and loaded on a flow cytometer (FACSCalibur) available for FL1 (annexin V) and FL2 (PI) bivariate analysis. Data from 20,000 cells/sample were collected, and the quadrants were set according to the population of viable, unstained cells in untreated samples. CellQuest analysis of the data was used to calculate the percentage of the cells in the respective quadrants.

### Enzymatic assay of caspase activity

Caspase activity was measured using a caspase fluorometric assay kit: Ac-Asp-Glu-Val-Asp-MCA for a caspase-3 substrate peptide, Ac-Leu-Glu-Val-Asp-AFC for a caspase-4 substrate peptide, Ac-Ile-Glu-Thr-Asp-MCA for a caspase-8 substrate peptide, and Ac-Leu-Glu-His-Asp-MCA for a caspase-9 substrate peptide. Cells were harvested before and after treatment with HUHS1015 and then centrifuged at 800*g* for 5 min at 4 °C. The pellet was incubated on ice in cell lysis buffer for 10 min and then centrifuged at 10,000*g* for 1 min at 4 °C. The supernatant was reacted with the fluorescently labeled tetrapeptide at 37 °C for 2 h. Fluorescence was measured at an excitation wavelength of 380 nm and an emission wavelength of 460 nm for caspase-3, caspase-8, and caspase-9 or at an excitation wavelength of 400 nm and an emission wavelength of 505 nm for caspase-4 with a fluorescence microplate reader (TECAN Infinite, Männedorf, Switzerland).

### Real-time reverse transcription-polymerase chain reaction (RT-PCR)

Before and after treatment with HUHS1015, total RNAs from cells were purified by an acid/guanidine/thiocyanate/chloroform extraction method using the Sepasol-RNA I Super kit (Nacalai, Kyoto, Japan). After purification, total RNAs were treated with RNase-free DNase I (2 units) at 37 °C for 30 min to remove genomic DNAs, and 10 μg of RNAs was resuspended in water. Then, random primers, dNTP, 10× RT buffer, and Multiscribe reverse transcriptase were added to an RNA solution and incubated at 25 °C for 10 min followed by 37 °C for 120 min to synthesize the first-strand cDNA. Real-time RT-PCR was performed using a SYBR Green Real-time PCR Master Mix (Takara Bio, Otsu, Japan) and the Applied Biosystems 7900 Real-Time PCR Detection System (ABI, Foster City, CA). Thermal cycling conditions were as follows: first step, 94 °C for 4 min; the ensuing 40 cycles, 94 °C for 1 s, 65 °C for 15 s, and 72 °C for 30 s. The expression level of each mRNA was normalized by that of GAPDH mRNA. Primers used for real-time RT-PCR are shown in Table 1.

**Table 1:** Primers used for real-time RT-PCR

FasL	
Sense	CACTACCGCTGCCACCCCTGA
Anti-sense	CATCATCTTCCCCTCCATCATCACC
Fas	
Sense	ATTATCGTCCAAAAGTGTTAATGCCCAA
Anti-sense	TGCACTTGGTGTTGCTGGTGAGTG
FADD	
Sense	GCCTGGGGAAGAAGACCTGTGTG
Anti-sense	CTGGCTTCCTGCTGGGTCTTCAC
TNFα	
Sense	CCTCCCCTGCCCCAATCCC
Anti-sense	GCTGGGCTCCGTGTCTCAAGG
TNFR1	
Sense	ATGCCGAAAGGAAATGGGTCAGG
Anti-sense	AAAATGACCAGGGGCAACAGCAC
TRADD	
Sense	GGGTTCCTTCTGCGGCTATTGCT
Anti-sense	TGAGTGAAACTGTAAGGGCTGGCTGTA
GAPDH	
Sense	GACTTCAACAGCGACACCCACTCC
Anti-sense	GGTCCACCACCCTGTTGCTGTAG

### Western blotting

Samples were loaded on 10 % (v/v) sodium dodecyl sulfate (SDS)-polyacrylamide gel electrophoresis (PAGE) and transferred to polyvinylidene difluoride membrane. After blocking with TBST (20 mM Tris, 150 mM NaCl, 0.1 % (v/v) Tween-20, pH 7.5) containing 5 % (w/v) of bovine serum albumin, blotting membrane was reacted with antibodies against FasL (Cell Signaling Technology, Inc., Danvers, MA, USA), Fas (Cell Signaling Technology), FADD (Cell Signaling Technology), tumor necrosis factor α (TNFα) (Cell Signaling Technology), TNFR1 (Santa Cruz Biotechnology, Inc., Dallas, Texas, USA), or TRADD (Santa Cruz Biotechnology) followed by a horseradish peroxidase (HRP)-conjugated anti-rabbit IgG or anti-goat IgG antibody. For β-actin detection, blotting membrane was reacted with an anti-β-actin antibody (SIGMA, Missouri, SL, USA) followed by an HRP-conjugated anti-mouse IgG antibody. Immunoreactivity was detected with an ECL kit (Invitrogen, Carlsbad, CA, USA) and visualized using a chemiluminescence detection system (GE Healthcare, Piscataway, NJ, USA). Protein concentrations for each sample were determined with a BCA protein assay kit (Thermo Fisher Scientific, Rockford, IL, USA).

### Inoculation of MKN45 cells

Nude BALB/c-*nu/nu* mice (male, 6 weeks) were obtained from Japan SLC, Inc. (Shizuoka, Japan). MKN45 cells (5 × 10^6^ cells) suspended in 200 μl of culture medium with 50 % (v/v) matrigel (BD Biosciences, San Jose, CA, USA) were subcutaneously inoculated into the right flank of mice under pentobarbital general anesthesia. HUHS1015, naftopidil, cisplatin, paclitaxel, and irinotecan were diluted with a physiological salt solution, and each solution was intraperitoneally injected twice a week from 1 week after inoculation. The longer (L) and shorter (S) lengths of inoculated tumors were measured using calipers, and tumor volume (V) was calculated according to the following equation: V = L × S^2^ × 1/2. Mice were killed on Day 33, and tumor was isolated and tumor weight was measured.

### Statistical analysis

Statistical analysis was carried out using unpaired *t* test, Dunnett’s test, and Fisher’s least significant difference (LSD) test. A *P* value of <0.05 was considered significant.

## Results

### HUHS1015 reduces cell viability for MKN28 and MKN45 cell lines more effectively than cisplatin

**Fig. 1:**
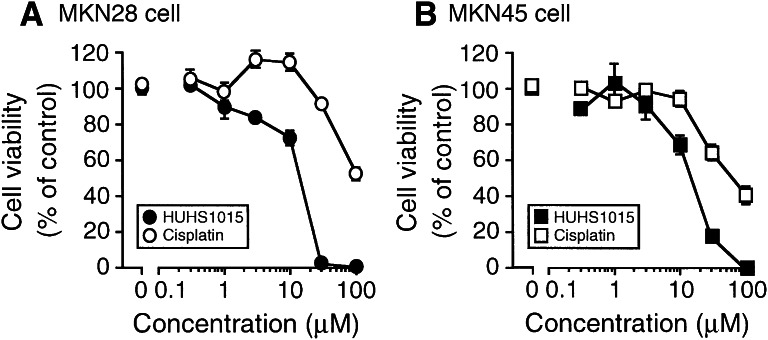
Effect of HUHS1015 on cell viability. MKN28 (**a)** and MKN45 cells (**b**) were treated with HUHS1015 or cisplatin at concentrations as indicated for 24 h, and cell viability was quantified with an MTT assay. Data represent the mean (±SEM) percentage of basal levels (MTT intensities of untreated cells) (*n* = 4 independent experiments)

HUHS1015 reduced MKN28 cell viability in a concentration (0.3–100 μM)-dependent manner (Fig. 1a). A similar effect was found in MKN45 cells (Fig. 1b). Cisplatin, a clinically used anticancer drug, also reduced cell viability for both MKN28 and MKN45 cell lines (Fig. 1a, b). There was a big difference in the potential between HUHS1015 and cisplatin; the much more beneficial effect was obtained with HUHS1015 (Fig. 1a, b).

### HUHS1015 induces caspase-independent and caspase-dependent apoptosis of MKN28 and MKN45 cells, respectively

**Fig. 2:**
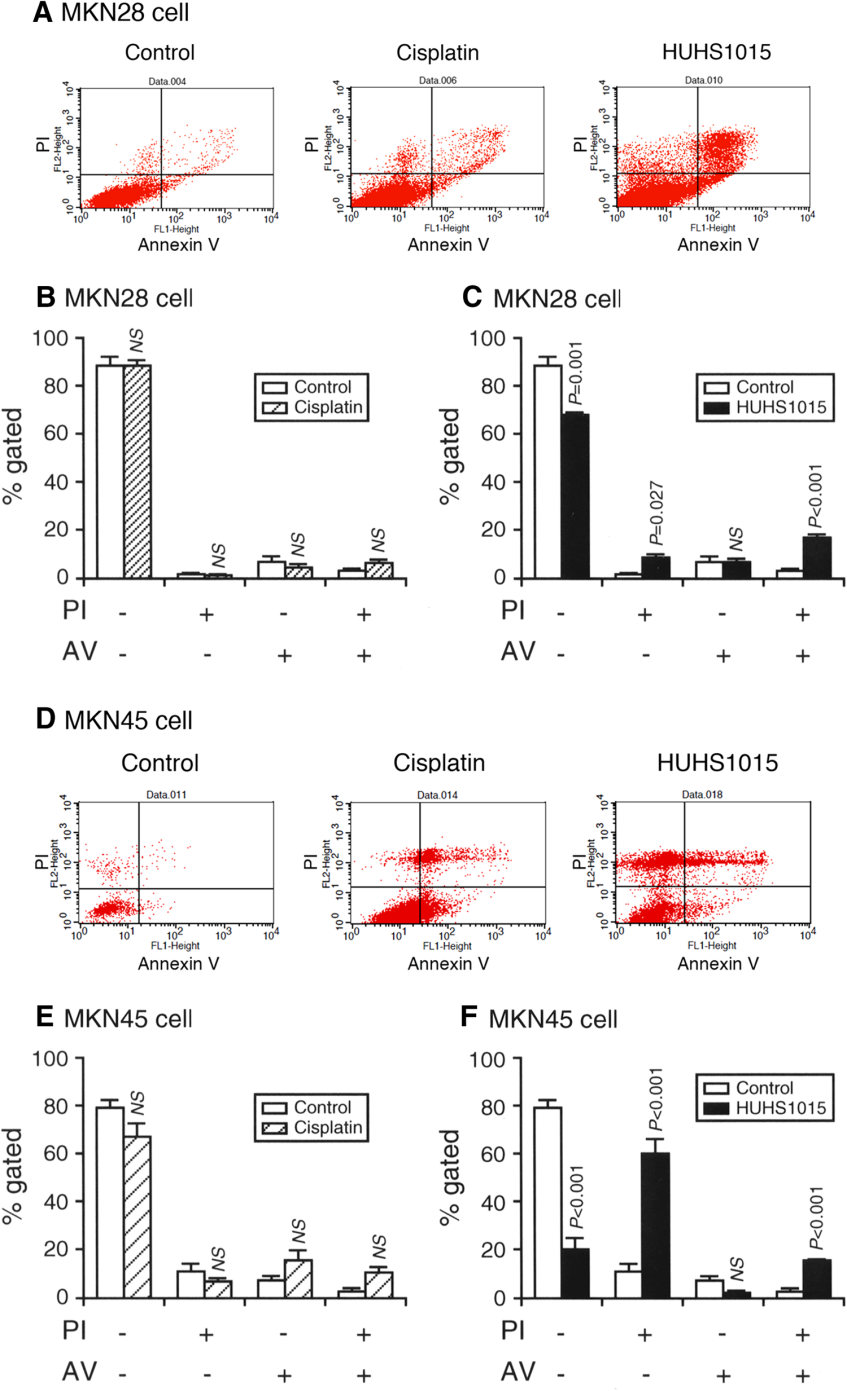
Flow cytometry using PI and annexin V-FITC (AV). MKN28 (**a**–**c**) and MKN45 cells (**d**–**f**) were not treated (control) and treated with cisplatin (15 μM) or HUHS1015 (15 μM) for 24 h. Typical profiles are shown in (**a**, **d**). In the graphs, each column represents the mean (±SEM) percentage of cells in four fractions against total cells (*n* = 4 independent experiments). *P* values, unpaired *t* test. *NS* not significant

In the flow cytometry analysis using PI and annexin V, PI is a marker of dead cells and annexin V, detecting externalized phosphatidylserine residues, is a marker of apoptotic cells [5]. In this assay, each population of PI-positive and annexin V-negative, PI-negative and annexin V-positive, or PI-positive and annexin V-positive cells corresponds to primary necrosis, early apoptosis, and late apoptosis/secondary necrosis, respectively [6]. HUHS1015 (15 μM) significantly increased the populations of PI-positive/annexin V-negative and PI-positive/annexin V-positive cells in both MKN28 (Fig. 2a, c) and MKN45 cells (Fig. 2d, f). This indicates that HUHS1015 induces both necrosis and apoptosis in MKN28 and MKN45 cells. In contrast, cisplatin (15 μM) had no significant effect on each population in MKN28 (Fig. 2a, b) and MKN45 cells (Fig. 2d, e). This suggests that cisplatin reduces viability of MKN28 and MKN45 cells by the mechanism distinct from that for HUHS1015, i.e., cisplatin-induced reduction of MKN28 and MKN45 cell viability is not due to necrosis and/or apoptosis.

**Fig. 3:**
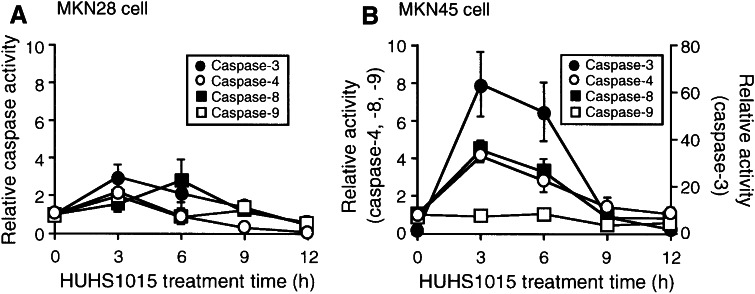
Enzymatic assay of caspase activity. Activities of caspase-3, caspase-4, caspase-8, and caspase-9 were assayed in MKN28 (**a**) and MKN45 cells (**b**) treated with HUHS1015 (100 μM) for periods of time as indicated. In the graphs, each *point* represents the mean (±SEM) ratio against basal caspase activities (0 h) (*n* = 4 independent experiments)

In the enzymatic caspase assay, no significant activation of caspase-3, caspase-4, caspase-8, and caspase-9 in MKN28 cells was obtained with HUHS1015 (100 μM) (Fig. 3a). This indicates that HUHS1015 induces caspase-independent apoptosis of MKN28 cells. In contrast, HUHS1015 (100 μM) significantly activated caspase-3, caspase-4, and caspase-8 in MKN45 cells (Fig. 3b). Notably, caspase-3 was activated to approximately 60 times of basal levels, while caspase-4 and caspase-8 were activated to about four times of basal levels (Fig. 3b). HUHS1015, accordingly, is likely to activate caspase-4 and caspase-8 followed by the effector caspase-3, to induce apoptosis.

### HUHS1015 upregulates expression of the TNFα mRNA and protein

Caspase-4 is activated in response to endoplasmic reticulum (ER) stress. Caspase-8, on the other hand, is activated through death receptor. The death receptor Fas, activated by FasL, recruits the adaptor protein Fas-associated death domain protein (FADD) to aggregate procaspase-8, that cleaves one other to initiate an active form of caspase-8, causing activation of the effector caspase-3 [7]. TNFα activates TNF receptor 1 (TNFR1), which forms a complex of TNFR1-associated death domain protein (TRADD)/FADD/procaspase-8, to activate caspase-8 followed by the effector caspase-3 [8]. We focused upon caspase-8 activation pathways and investigated whether HUHS1015 affects expression of death receptor-related molecules in MKN45 cells.

**Fig. 4:**
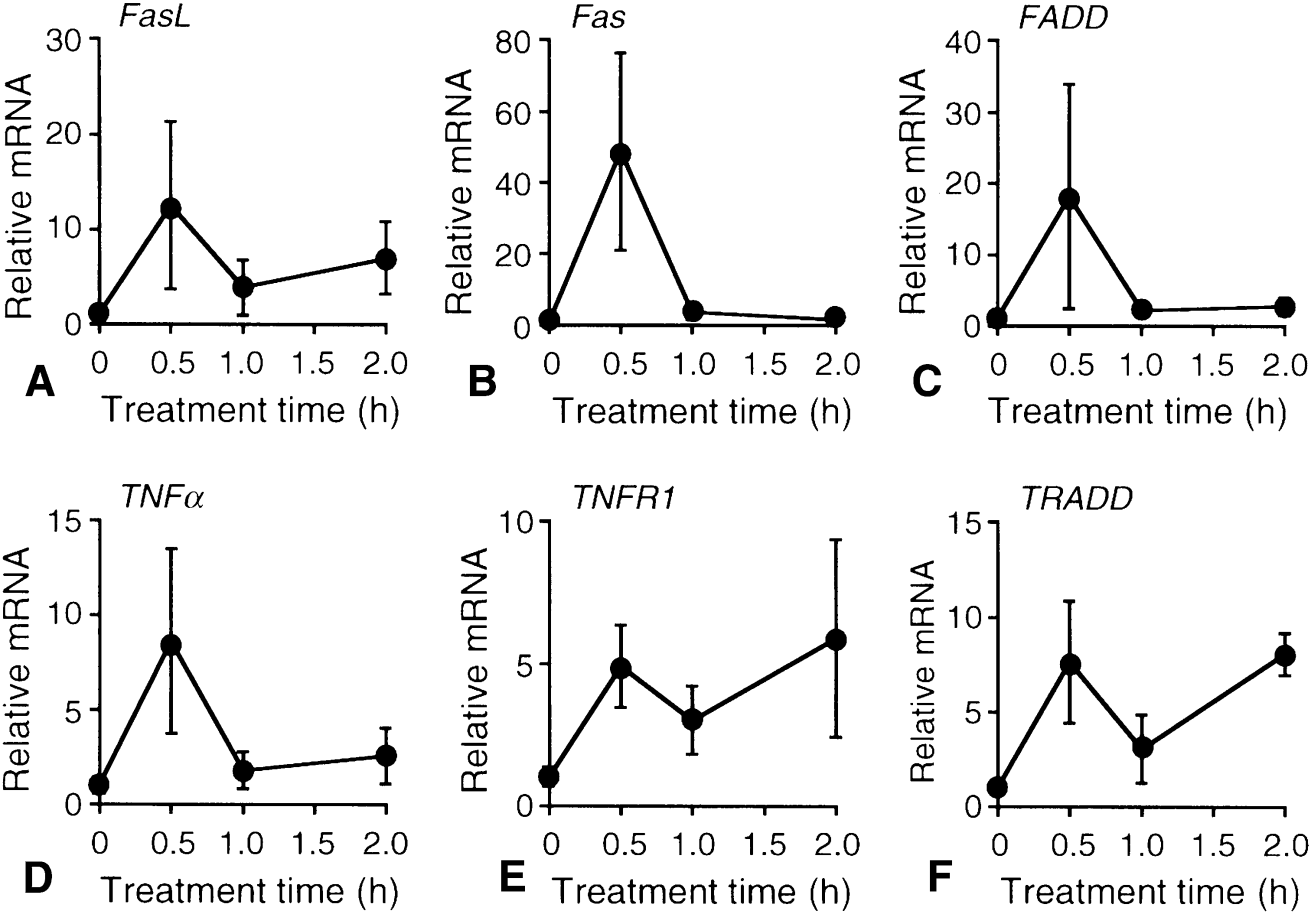
Real-time RT-PCR analysis. MKN45 cells were treated with HUHS1015 (100 μM) for periods of time as indicated, and then, real-time RT-PCR was carried out. The mRNA quantity for FasL (**a**), Fas (**b**), FADD (**c**), TNFα (**d**), TNFR1 (**e**), and TRADD (**f**) was calculated from the standard *curve* made by amplifying different amount of the GAPDH mRNA and normalized by regarding the average of independent basal mRNA quantity at 0 h as 1. In the graphs, each *point* represents the mean (±SEM) ratio relative to basal mRNA levels (*n* = 4 independent experiments)

In the real-time RT-PCR analysis, HUHS1015 (100 μM) upregulated expression of all the mRNAs examined here for FasL, Fas, FADD, TNFα, TNFR1, and TRADD at 0.5-h treatment in MKN45 cells (Fig. 4a–f).

**Fig. 5:**
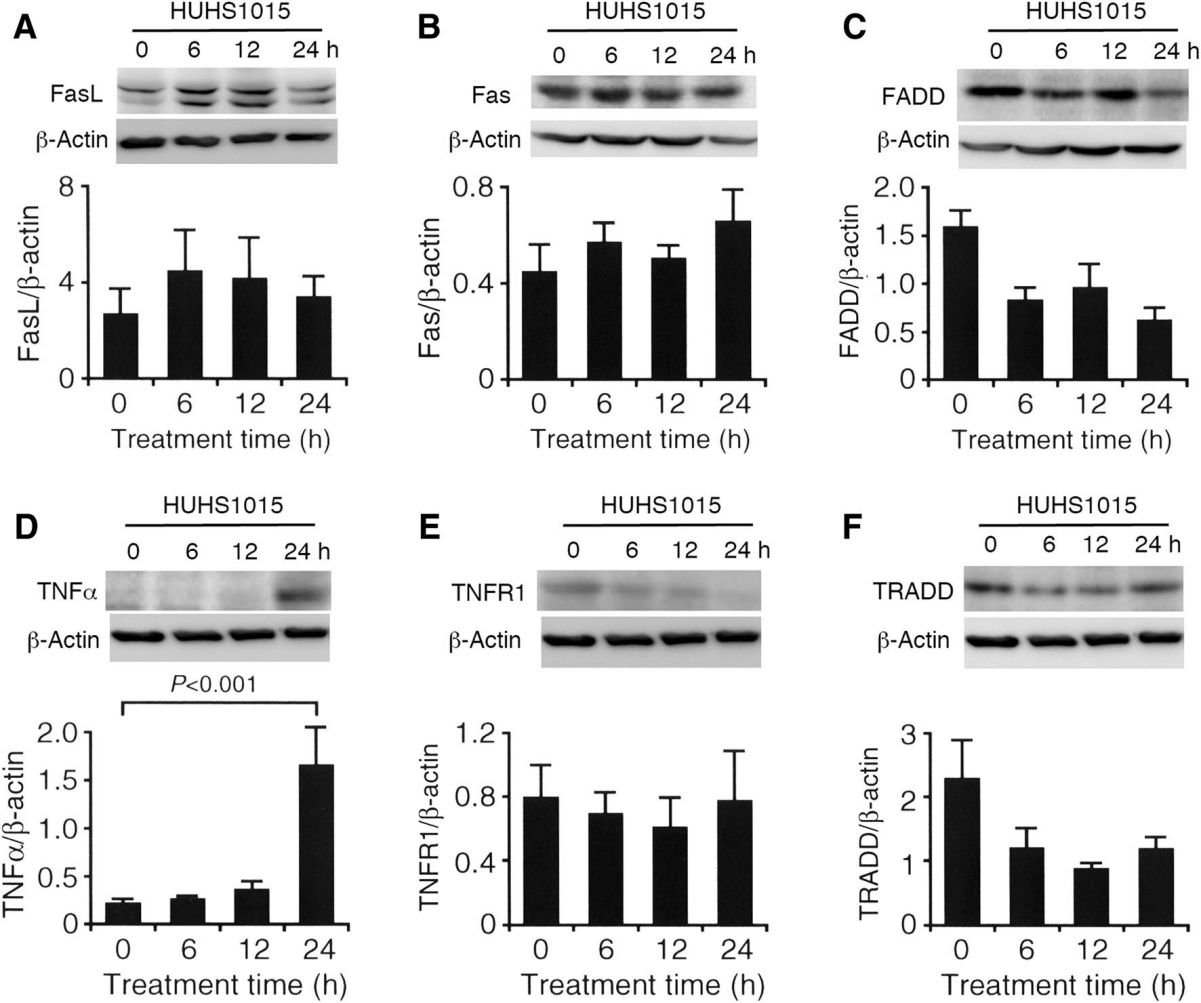
Western blot analysis. MKN45 cells were treated with HUHS1015 (100 μM) for periods of time as indicated, and Western blotting was carried out. The signal intensity for FasL (**a**), Fas (**b**), FADD (**c**), TNFα (**d**), TNFR1 (**e**), or TRADD protein (**f**) was normalized by that for β-actin. In the graphs, each column represents the mean (±SEM) normalized intensity for each protein (*n* = 4 independent experiments). *P* value, Dunnett’s test

In the Western blot analysis, HUHS1015 (100 μM) upregulated expression of TNFα protein at 24-h treatment in MKN45 cells (Fig. 5d), although expression of proteins for FasL, Fas, FADD, TNFR1, and TRADD was not increased (Fig. 5a–c, e, f). Collectively, these results suggest that HUHS1015 upregulates expression of the TNFα mRNA and protein in MKN45 cells, thereby causing activation of TNFR1 and caspase-8. This may contribute at least in part to HUHS1015-induced caspase-3 activation.

### HUHS1015 suppresses proliferation of MKN45 cells

**Fig. 6:**
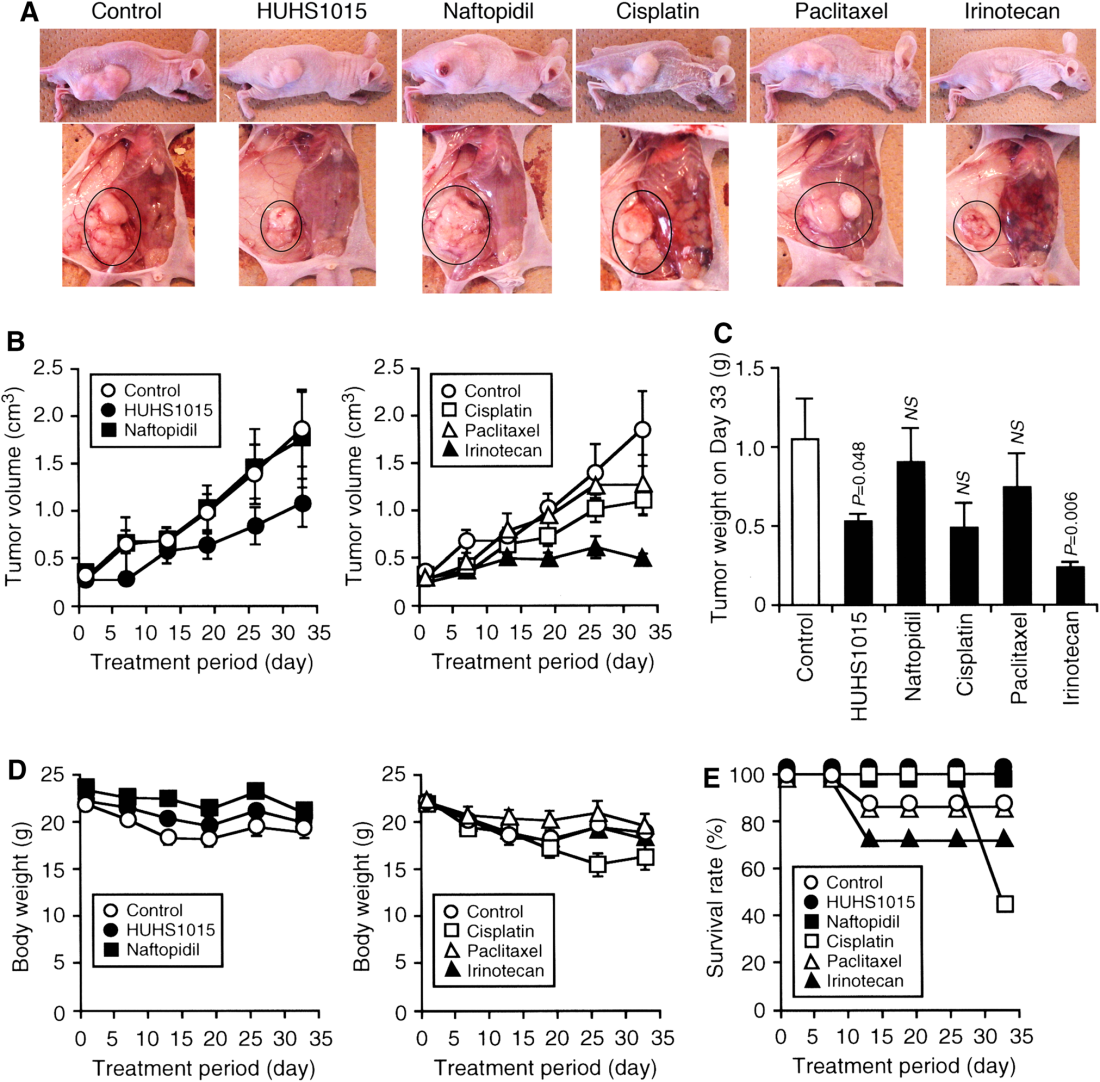
HUHS1015-induced suppression of MKN45 cell proliferation in mice. MKN45 cells were subcutaneously inoculated into the flank of mice, and a week later saline (control), HUHS1015 (9.16 mg/kg), naftopidil (9.81 mg/kg), cisplatin (7.50 mg/kg), paclitaxel (21.35 mg/kg), or irinotecan (15.58 mg/kg) was intraperitoneally injected twice a week. At the beginning of experiments, seven independent mice were used for each trial. **a** Tumor outward appearance on Day 33 and autopsy (*black round*). **b** Time-course change of tumor volume (the mean ± SEM). **c** Tumor weight on Day 33 (the mean ± SEM). *P* values as compared with control tumor volume, Fisher’s LSD test. *NS* not significant. **d** Time-course change of body weight (the mean ± SEM). **e** Survival rate at the periods of time as indicated (the mean ± SEM)

We finally examined the effect of HUHS1015 on tumor growth using mice inoculated with MKN45 cells. HUHS1015 (9.16 mg/kg, ~25 μM), naftopidil (9.81 mg/kg, ~25 μM), cisplatin (7.50 mg/kg, ~25 μM), paclitaxel (21.35 mg/kg, ~25 μM), or irinotecan (15.58 mg/kg, ~25 μM) was intraperitoneally injected twice a week from 1 week after inoculation. Tumor grew day by day, and tumor volume for saline-injected control mice reached six times on Day 33 (Fig. 6a, b). HUHS1015 clearly inhibited an increase in the tumor volume as compared with that for control mice, while no inhibition of tumor growth was found with naftopidil (Fig. 6a, b). Tumor weight for mice treated with HUHS1015 on Day 33 was significantly lower than that for control mice (Fig. 6c). The anticancer drugs cisplatin, paclitaxel, and irinotecan also inhibited tumor growth, with the order of the potential: irinotecan ≫ cisplatin > paclitaxel (Fig. 6a, b). Tumor weight for mice treated with irinotecan on Day 33 was much lower as compared with that for control mice, but otherwise no significant effect on tumor weight was obtained with naftopidil, cisplatin, or paclitaxel (Fig. 6c). Marked loss of body weight was found in mice treated with cisplatin, while there was no remarkable body weight loss in the remaining groups (Fig. 6d). The survival rate throughout experiments was 100 % for mice treated with HUHS1015 and naftopidil, 86 % for ones treated with saline (control) and paclitaxel, 71 % for ones treated with irinotecan, and 43 % for ones treated with cisplatin (Fig. 6e). Taken together, these results indicate that HUHS1015 could effectively suppress proliferation of MKN45 gastric cancer cells, with the higher survival rate.

## Discussion

The results of the present study demonstrate that HUHS1015 reduces cell viability for both MKN28 human well-differentiated gastric adenocarcinoma cell line and MKN45 human poorly differentiated gastric adenocarcinoma cell line in a concentration (0.3–100 μM)-dependent manner, more effectively than cisplatin. Cisplatin is a platinum-compound chemotherapy drug to act as an alkylating agent and used for treatment of testicular, bladder, ovarian, and several other cancers such as gastric and lung cancers [9]. In the flow cytometry analysis using PI and annexin V, HUHS1015 significantly increased the populations of PI-positive/annexin V-negative and PI-positive/annexin V-positive cells in MKN28 and MKN45 cells, indicating that HUHS1015 induces necrosis and apoptosis in both cell types. No such effect, however, was found with cisplatin. This implies that cisplatin reduces cell viability for MKN28 and MKN45 cells by the mechanism independent of necrosis and/or apoptosis.

HUHS1015 apparently activated caspase-3, caspase-4, and caspase-8 in MKN45 cells, while no activation of caspase-3, caspase-4, caspase-8, and caspase-9 was obtained in MKN28 cells. This implies that HUHS1015 induces caspase-independent apoptosis of MKN28 cells and caspase-dependent apoptosis of MKN45 cells; in other words, HUHS1015 induces apoptosis of MKN28 and MKN45 cells by the different mechanisms.

Caspase-3 is activated as an effector caspase of caspase-4 or caspase-8, to induce apoptosis. Accumulation of unfolding or misfolding protein in the ER causes ER stress, which triggers caspase-4 activation. It is presently far from understanding how HUHS1015 activates caspase-4. Death receptors such as Fas and TNFR1, on the other hand, participate in the activation of caspase-8. FasL activates Fas, which forms a complex with FADD and procaspase-8, to activate caspase-8 [7]. TNFα activates TNFR1, which forms a complex with TRADD, FADD, and procaspase-8, to activate caspase-8 [8]. HUHS1015 upregulated expression of mRNAs for FasL, Fas, FADD, TNFα, TNFR1, and TRADD at 0.5-h treatment in MKN45 cells. Among them, a significant increase in the expression of TNFα protein alone was induced by 24-h treatment with HUHS1015. These results suggest that HUHS1015 upregulates expression of the TNFα mRNA and protein in MKN45 cells, thereby causing caspase-8 activation through TNFR1. This may account at least in part for HUHS1015-induced caspase-3 activation in MKN45 cells. In spite of only fourfold activation of caspase-4 and caspase-8, nearly 60-fold activation of caspase-3 was induced by HUHS1015. This raises the possibility that an additional unknown mechanism underlies HUHS1015-induced caspase-3 activation, regardless of caspase-4 and caspase-8 activation.

In the in vivo experiments using mice inoculated with MKN45 cells, HUHS1015 obviously suppressed tumor growth, but naftopidil otherwise had no significant effect. This indicates that the naftopidil analogue HUHS1015 is more effective on gastric cancer cells than naftopidil by itself. The anticancer drugs cisplatin, paclitaxel, and irinotecan also suppressed tumor growth, and the order of the potential for all the investigated compounds was irinotecan ≫ HUHS1015·cisplatin > paclitaxel > naftopidil. The survival rate for HUHS1015 treatment throughout experiments was 100 %, while that for cisplatin treatment was 43 %, in association with severe weight loss. Paclitaxel [10, 11], a mitotic inhibitor, and irinotecan [12], which is made from a type of plant alkaloid known as a topoisomerase I inhibitor, are used in chemotherapy for a variety of cancers including gastric cancer. The survival rate for paclitaxel and irinotecan treatment throughout experiments was 86 and 71 %, respectively. Taken together, these results indicate that HUHS1015 is useful for treatment of gastric cancer, with the survival rate higher than that for the anticancer drugs cisplatin, paclitaxel, and irinotecan.

In the present study, the anticancer effect of drugs was assessed using a subcutaneous implantation model of stomach cancer. Tumors subcutaneously implanted are grown in a very different micro-environment from the tissue of origin of the tumors, which may result in the lack of metastases and the altered drug responses. To address these problems, a surgical orthotopic implantation (SOI) model has been developed [13, 14]. We are currently attempting to obtain the more physiological effect of HUHS1015 on gastric cancer using the SOI model.

In conclusion, the results of the present study show that HUHS1015 induces caspase-independent and caspase-dependent apoptosis of MKN28 and MKN45 gastric cancer cells, respectively, and suppresses tumor growth in mice inoculated with MKN45 cells, with the higher survival rate as compared with that for anticancer drugs examined here. HUHS1015 thus may offer new hope for treatment of gastric cancer.

## Abbreviations

HUHS1015: 1-[2-(2-Methoxyphenylamino)ethylamino]-3-(naphthalene-1-yloxy)propan-2-ol
MTT: 3-(4,5-Dimethyl-2-thiazolyl)-2,5-diphenyl-2H-tetrazolium bromide
PI: Propidium iodide
RT-PCR: Reverse transcription-polymerase chain reaction
TNFα: Tumor necrosis factor α
HRP: Horseradish peroxidase
LSD: Least significant difference
ER: Endoplasmic reticulum
FADD: Fas-associated death domain protein
TNFR1: TNF receptor 1
TRADD: TNFR1-associated death domain protein
